# Vaccine impact on antimicrobial resistance to inform Gavi, the Vaccine Alliance’s 2018 Vaccine Investment Strategy: report from an expert survey

**DOI:** 10.12688/f1000research.20100.1

**Published:** 2019-09-24

**Authors:** Maya Malarski, Mateusz Hasso-Agopsowicz, Adam Soble, Wilson Mok, Sophie Mathewson, Johan Vekemans

**Affiliations:** 1 Policy, Vaccines & Sustainability, Gavi, the Vaccine Alliance, Le Grand-Saconnex, 1218, Switzerland; 2 Immunization, Vaccines, and Biologicals, World Health Organization, Geneva, 1211, Switzerland; 3 Vaccine Supply & Demand, Vaccines & Sustainability, Gavi, the Vaccine Alliance, Le Grand-Saconnex, 1218, Switzerland

**Keywords:** Vaccines, antimicrobial resistance

## Abstract

**Background:** While the rise of antimicrobial resistance (AMR) has been recognised as a major public health problem, the value of vaccines to control AMR is poorly defined. This expert survey was launched with the aim of informing the 2018 Vaccine Investment Strategy through which Gavi, the Vaccine Alliance prioritises future vaccine funding. This exercise focused on both vaccines currently supported by Gavi and under consideration for future funding.

**Methods:** The relative importance of pre-defined criteria as drivers of overall value of vaccines as a tool/ intervention to control AMR was assessed by 18 experts: prevention of mortality and morbidity due to resistant pathogens, antibiotic use prevented, societal impact, ethical importance and sense of urgency. For each vaccine, experts attributed scores reflecting the estimated value for each criterion, and overall value relative to AMR was derived from the value assigned to each criterion and their relative importance for each vaccine.

**Results:** Mortality, morbidity due to targeted resistant pathogens, and antibiotic use prevented were considered the most important determinants of overall value. Pneumococcal, typhoid and malaria vaccines were assigned highest value relative to antimicrobial resistance. Intermediate value was estimated for specific rotavirus, cholera, respiratory syncytial virus (RSV), influenza, dengue, measles, meningitis and *Haemophilus influenza *type b- (Hib-) containing pentavalent vaccines. Lowest value relative to AMR was estimated for Japanese encephalitis, hepatitis A, yellow fever, rabies and human papilloma virus vaccine.

**Conclusions:** In the future, more evidence-based, data-driven, robust methodologies should be developed to guide coordinated, rational decision making on priority actions aimed at strengthening the use of vaccines against AMR.

## Abbreviations

Antimicrobial resistance (AMR) ; respiratory syncytial virus (RSV) ; Haemophilus influenza type b (Hib) ; World Health Organization (WHO) ; pneumococcal conjugate vaccine (PCV) ; inactivated polio vaccine (IPV) ; Vaccine Investment Strategy (VIS) ; diphtheria-tetanus-pertussis (DTP) ; typhoid conjugate vaccine (TCV) ; World Organisation for Animal Health (OIE).

## Highlights

- Vaccines contribute to combat the growing threat of antimicrobial resistance
- A survey was undertaken to assess expert opinion about the value relative to antimicrobial resistance of potential vaccine investments considered by Gavi, the Vaccine Alliance
- Each expert assigned vaccine value by assigning a score to a set of pre-defined criteria, weighted for their relative importance: prevention of mortality and morbidity due to resistant pathogens, antibiotic use prevented, societal impact, ethical importance and sense of urgency
- Experts considered mortality, morbidity due to targeted resistant pathogens, and antibiotic use prevented as the most important determinants of overall value
- Pneumococcal, typhoid and malaria vaccines were assigned highest value relative to antimicrobial resistance
- This exercise will help shaping future evidence-based assessment of the public value of vaccines to contribute to control of antimicrobial resistance

## Background

Antimicrobial resistance (AMR) constitutes a major global health threat. Each year, an estimated 700,000 deaths result from infections with pathogens resistant to antimicrobial drugs, and this toll is expected to rise to 10 million by 2050 ^1^. There is strong political momentum around the need to prioritise prevention of AMR pathogens in the global health agenda, as highlighted by the high-level meeting of the United Nations General Assembly on antimicrobial resistance ^2^ and the Global Action Plan on Antimicrobial Resistance ^3^. Recently, the World Health Organization (WHO) issued a list of priority pathogens against which new antibiotics should be developed ^4^. With the expanding burden related to AMR, diminishing treatment options for a number of bacterial diseases, and a small pipeline of potential new therapeutics ^5^, vaccines are increasingly recognised as an important complementary tool in controlling AMR ^6– 10^.

Vaccines have the potential to impact antibiotic resistance in several ways. Vaccines targeting a bacterial pathogen can reduce the vaccinated individual’s risk of an infection, not only protecting that individual but also possibly preventing further transmission of potential resistant strains. Both bacterial and viral vaccines can reduce the incidence of illnesses that prompt antibiotic use. This helps to reduce the selective pressure driving emergence of resistance on the targeted pathogen as well as the whole microbiome of the host. Some vaccines can also reduce bacterial carriage and the size of the pathogen population in the host, thereby reducing the risk of emergence of resistance and spread ^11^. By decreasing the incidence of vaccine-preventable diseases, vaccines reduce care-seeking behaviour, such as attendance in health facilities, and thereby exposure to AMR pathogens ^12^.

Evidence which demonstrates the impact of some vaccines against AMR is available ^10^. Within five years of the first introduction of pneumococcal conjugate vaccine (PCV) in the United States, prevalence of pneumococcal multidrug-resistant strains reduced by 57%, and the incidence of multidrug-resistant invasive pneumococcal disease decreased by 84% in children under two years old ^13^. A similar post-introduction study in South Africa found that the incidence of invasive pneumococcal disease caused by PCV7 serotypes decreased by 85% in children not infected with human immunodeficiency virus ^14^. A study estimated that global and widespread use of PCV could reduce the amount of antibiotics used for pneumonia patients by 47%, the equivalent of 11.4 million antibiotics days globally ^5^. Similarly, introduction of *Haemophilus influenzae* type b (Hib) vaccines in the 1980s led to demonstrated reductions in the prevalence of Hib drug-resistant strains ^10, 16^. In Canada, an ecological study suggested that universal, free introduction of seasonal influenza vaccination in certain regions of the country led to a significant reduction of antibiotic prescriptions ^17^. This remains a limited body of evidence, and the need for more research and modelling evaluation of vaccine impact on AMR has been clearly expressed ^18^.

Gavi, the Vaccine Alliance was established in 2000 with the goal of creating equal access to new and underused vaccines for those living in lower-income countries. Since its inception, the Alliance has played a critical role in ensuring access to these vaccines, currently supporting vaccines that protect against 16 pathogens and contributing to the immunisation of more than 700 million children ^19^. Every five years Gavi reviews its Vaccine Investment Strategy (VIS), to identify new vaccines and other immunisation products of most importance to Gavi-supported countries. The VIS sets new priorities for Gavi’s vaccine support programmes through an in-depth analysis of impact, cost, value and programmatic feasibility. Prioritised investments are included in Gavi’s portfolio. In 2017, Gavi commenced the development of its latest VIS, covering the 2021–2025 strategic period. Under consideration were 12 vaccine candidates and other immunisation products for endemic diseases, support for pandemic influenza preparedness, and inactivated polio vaccine (IPV) support post-2020 ^20^.

To inform the prioritisation of Gavi’s potential future investments, an evaluation framework was developed. Various criteria including health impact, value for money, equity and social protection impact, economic impact and global health security were considered, along with other secondary criteria. This was the first time an indicator on “Impact of vaccination on AMR” was included in the evaluation framework for prioritisation of vaccine investments. The indicator sits within the broader “Global Health Security” criterion, which also separately considers the impact of vaccination on diseases with epidemic potential ^20^. This article reports the results of an expert consultation that was used to assess the comparative value of potential vaccines against AMR, as one component of the wider prioritisation process.

## Methods

In view of the limited evidence available and time constraints to develop a more comprehensive analysis method for contribution to the VIS 2018, an approach based on expert opinion was selected through a collaboration between the Gavi Secretariat and the WHO Initiative for Vaccine Research. Both the current portfolio of Gavi-supported vaccines and those selected as part of the VIS 2018 exercise were considered, as detailed in Table 1. Previous Gavi investment prioritisation decisions did not formally consider the potential contribution of vaccines to control AMR.

**Table 1.** Vaccines and associated vaccination strategies evaluated. DTP (Diphtheria-, pertussis and tetanus); Penta (pentavalent vaccine); Td (tetanus and diphtheria booster vaccine); Meningitis ACWY or ACWXY (meningitis vaccine against A, C, W, Y strains or A, C, W, X, Y strains); RSV (respiratory syncytial virus); mAb (monoclonal antibody).

	Vaccine	Vaccination strategy	Target age group/Population
**Candidate Vaccines in VIS 2018**	**Cholera (oral)**	Campaigns every 3–5 years	>1 year old
	**Dengue**	Routine Immunisation	Product dependant: 2, 4 or 9 year olds
	**Diphtheria-, pertussis- and tetanus (DTP)-** **containing vaccine boosters**	Routine immunisation with 3 boosters	1 year old (DTP or Penta), 5 year old (Td) and 10 year old (Td)
	**Hepatitis A**	Routine Immunisation	12 months
	**Hepatitis B birth dose**	Routine Immunisation	Within 24hrs of birth
	**Maternal Influenza**	Routine Immunisation	Maternal immunisation (24–36 weeks gestation) against seasonal influenza
	**Malaria (RTS,S)**	Routine Immunisation	1 ^st^ & 3 ^rd^ year of life
	**Meningitis ACWY or ACWXY (conjugate)**	Routine and mass preventive campaigns	Routine: 1 ^st^ year of life and/or 2 ^nd^ year of life Campaign: 1–29 year olds or 5–14 year olds
	**Rabies vaccine**	Post-exposure prophylaxis	Bite victims seeking treatment
	**Rabies immunoglobulin**	Post-exposure prophylaxis	Patients with severe bites
	**Respiratory Syncytial Virus (RSV)** **monoclonal antibody (mAb)**	Routine Immunisation	All infants
	**Maternal Respiratory Syncytial Virus (RSV)** **vaccine**	Routine Immunisation	Maternal immunisation (24–36 weeks gestation)
**Current Gavi-supported vaccines**	**Human Papillomavirus**	Routine cohort with multi-age cohort at introduction	Girls aged 9–14
	**Japanese Encephalitis**	Routine with one-time catch-up campaign	Routine: 12 month olds Catch-up: 9 months to 14 years
	**Measles**	Routine and follow-up campaign	Routine: Infants in 1 ^st^ and 2 ^nd^ year of life Follow-up: 9 months to 59 months
	**Measles & Rubella**	Routine and catch-up campaign	Routine: Infants in 1 ^st^ and 2 ^nd^ year of life Catch-up: 9 months to 14 years Follow-up: 9 months to 59 months
	**Meningitis A**	Routine and catch-up campaign or mass preventative campaign	Routine: 9–18 months Catch-up: cohorts not captured by previous campaign or routine Preventative campaign: 1–29 year olds
	**Pentavalent**	Routine Immunisation	1 ^st^ year of life
	**Pneumococcal conjugate vaccine**	Routine Immunisation	1 ^st^ year of life
	**Rotavirus**	Routine Immunisation	1 ^st^ year of life
	**Typhoid conjugate vaccine**	Routine and catch-up campaign	1 ^st^ year of life
	**Yellow Fever**	Routine and mass preventative campaign	Routine: 9 months in Africa and 12 months in Americas Preventative campaign: at risk populations

Based on internal, institutional discussions, the list of criteria used to assess the value of vaccines against AMR was generated. Six criteria were identified, as presented in Table 2.

**Table 2.** Evaluation Criteria.

Name of criterion	Definition of criterion
Mortality	**Mortality** due to resistant pathogens that will be prevented by the vaccine through a direct effect (resistance within the vaccine-targeted organism)
Morbidity	**Morbidity** due to resistant pathogens that will be prevented by the vaccine through a direct effect (resistance within the vaccine-targeted organism)
Societal impact	**Societal impact** from vaccine-targeted resistant pathogens (considering burden on health systems and economic dimensions)
Ethical importance	**Ethical importance** of vaccine-targeted resistant pathogens as sources of inequity and social exclusion
Antibiotic use prevented by the vaccine	**Antibiotic use prevented by the vaccine** (considering the diagnoses and fraction of disease syndromes that may be reduced by the considered vaccine, leading to prevention of antibiotic use according to standard recommendations, or not, and based on the usual treatment duration and spectrum of action of frequently used antibiotics for the associated diagnoses or syndromic presentations)
Time trend and sense of urgency	**Time trend and sense of urgency** related to AMR threat due to vaccine-targeted pathogen (consider trends in incidence and ability to treat): consider the therapeutic options in the coming 10 years horizon, the general transmissibility

Experts were identified from high impact AMR related research and publications; from the list of participants of AMR related meetings; and through a consultation with other experts and funders. They were eligible to participate in the study if they had a relevant expertise in AMR across the fields of infectious diseases, clinical microbiology, vaccinology, public health, epidemiology. Global geographic representation and gender balance were sought. Participants were included in the survey if they provided a complete set of responses. Background information (available as the extended data ^32^) provided to each expert included a basic description about the vaccine investments considered and global disease burden data ^21^.

On the 1 ^st^ of February 2018 experts received an email and an Excel file with an introduction and background information tabs that explained the process of scoring each vaccine and pathogen (extended data ^32^) and were asked to respond by the 13 ^th^ of February. The file explained that experts are expected to weigh the relative importance of each criterion, by distributing 100% points across the six criteria. The experts were then asked to assign a score between 1 and 10 (where 1 = least effect of vaccine in future and 10 = greatest effect of vaccine in future) to each criterion, for all vaccines included in the assessment.

The average weight per criterion was calculated in Excel and expressed using lower and upper quartile ranges, minimum and maximum values, means and medians, and outliers defined as over 1.5*interquartile range from the 1st or 3rd quartile. The vaccine scores (vaccine impact on AMR) for each criterion were calculated in Excel and expressed using average scores. The overall impact of vaccines on AMR (aggregate vaccine scores for all criteria) was calculated in Excel by multiplying the average weight per criterion and the vaccine scores (vaccine impact on AMR) assigned by each expert to different criteria. It was expressed using lower and upper quartile ranges, minimum and maximum values, means and medians, and outliers defined as over 1.5*interquartile range from the 1st or 3rd quartile.

## Results

The survey was launched in February 2018. Of the 26 experts approached to participate, 18 completed the survey and all responses were complete. Experts were from the following countries: Australia, India, Republic of Korea, Republic of South Africa, Spain, Sweden, Switzerland, the United Kingdom and the United States. Ten experts were from research institutions, four from international organisations, two from public health institutes and two were independent (see underlying data ^33^).

**Figure 1.**
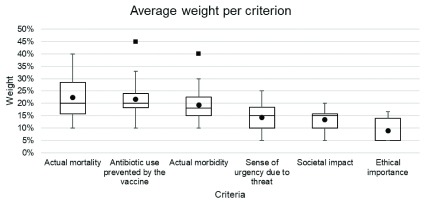
The weighting assigned to each criterion by experts against which vaccine candidates were assessed. Lower error bar= minimum value; lower bound of the box= lower quartile range; black line inside the box=median; upper bound of the box=upper quartile range; upper error bar= maximum value; circle=mean; square = outlier (defined as over 1.5*interquartile range from the 1 ^st^ or 3 ^rd^ quartile).

Figure 1 shows the distribution of weights assigned to each of the criteria by the experts, out of a total of 100%. Higher importance was attributed to mortality (median 20%, interquartile range [IQR] 16-29%), antibiotic use prevented by the vaccine (20%, IQR 18-24%) and morbidity (18%, IQR 15-23%). Less importance was attributed to the sense of urgency due to AMR threat (15%, IQR 10-19%), societal impact (15%, IQR 10-16%) and ethical importance (5%, IQR 5-14%).

**Figure 2.**
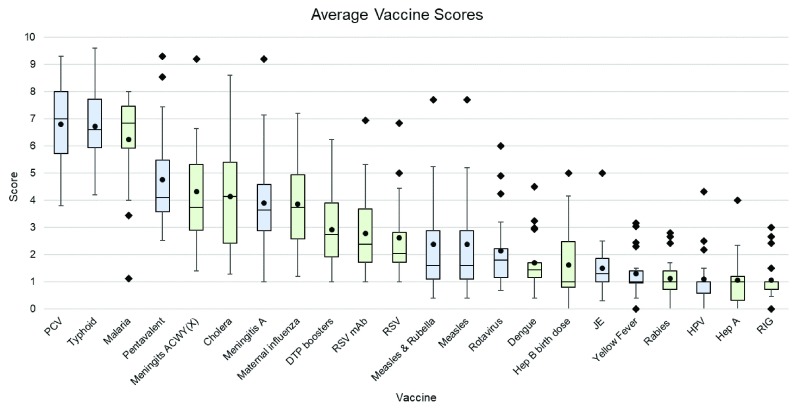
Overall vaccine impact on AMR as estimated by experts, boxplots. 1= least effect of vaccine and 10 = greatest effect of vaccine on AMR Lower error bar= minimum value; lower bound of the box= lower quartile range; black line inside the box=median; upper bound of the box=upper quartile range; upper error bar= maximum value; circle=mean; square = outlier (defined as over 1.5*interquartile range from the 1st or 3rd quartile) Blue shading = Gavi portfolio vaccines; Green shading = VIS candidate vaccines PCV – pneumococcal conjugate vaccine; Pentavalent vaccine (diphtheria, pertussis, tetanus, hepatitis B and Haemophilus influenzae type B); DTP – diphtheria, tetanus & pertussis; RSV – Respiratory syncytial virus; mAb - monoclonal antibodies; Hep B – Hepatitis B; JE – Japanese encephalitis; HPV – Human papillomavirus; Hep A – Hepatitis A; RIG – Rabies immunoglobulin.

Figure 2 presents the overall expert-estimated impact on AMR for the considered vaccine investments. Within the current Gavi portfolio, the three investments that had the highest estimated impact on AMR were those related to pneumococcal conjugate (median impact 7.00, IQR 5.71–8.00), typhoid conjugate (6.60, IQR 5.95–7.73) and pentavalent (diphtheria, pertussis, tetanus, hepatitis B and Hib), (4.10, IQR 3.59–5.47) vaccines. Among the potential vaccine investments considered as part of the VIS 2018 exercise, the three vaccines with the highest predicted impact on AMR were vaccines for malaria (6.85, IQR 5.93–7.46), cholera (4.15, IQR 2.43–5.40) and meningitis ACWY(X), (3.75, IQR 2.90–5.33). In contrast, the three vaccines currently funded by Gavi that received the lowest score for estimated impact on AMR were vaccines for Japanese encephalitis (1.30, IQR 1.00–1.86), yellow fever (1.00, IQR 0.95–1.40) and human papillomavirus (HPV; 1.00, 0.59–1.00). Among the vaccine investments considered as part of the VIS 2018 exercise, those with the lowest estimated AMR impact were vaccines for rabies (1.00, IQR 0.73–1.40), hepatitis A (1.00, IQR 0.33–1.20) and rabies immunoglobulin (1.00, IQR 0.73–1.00).

**Figure 3.**
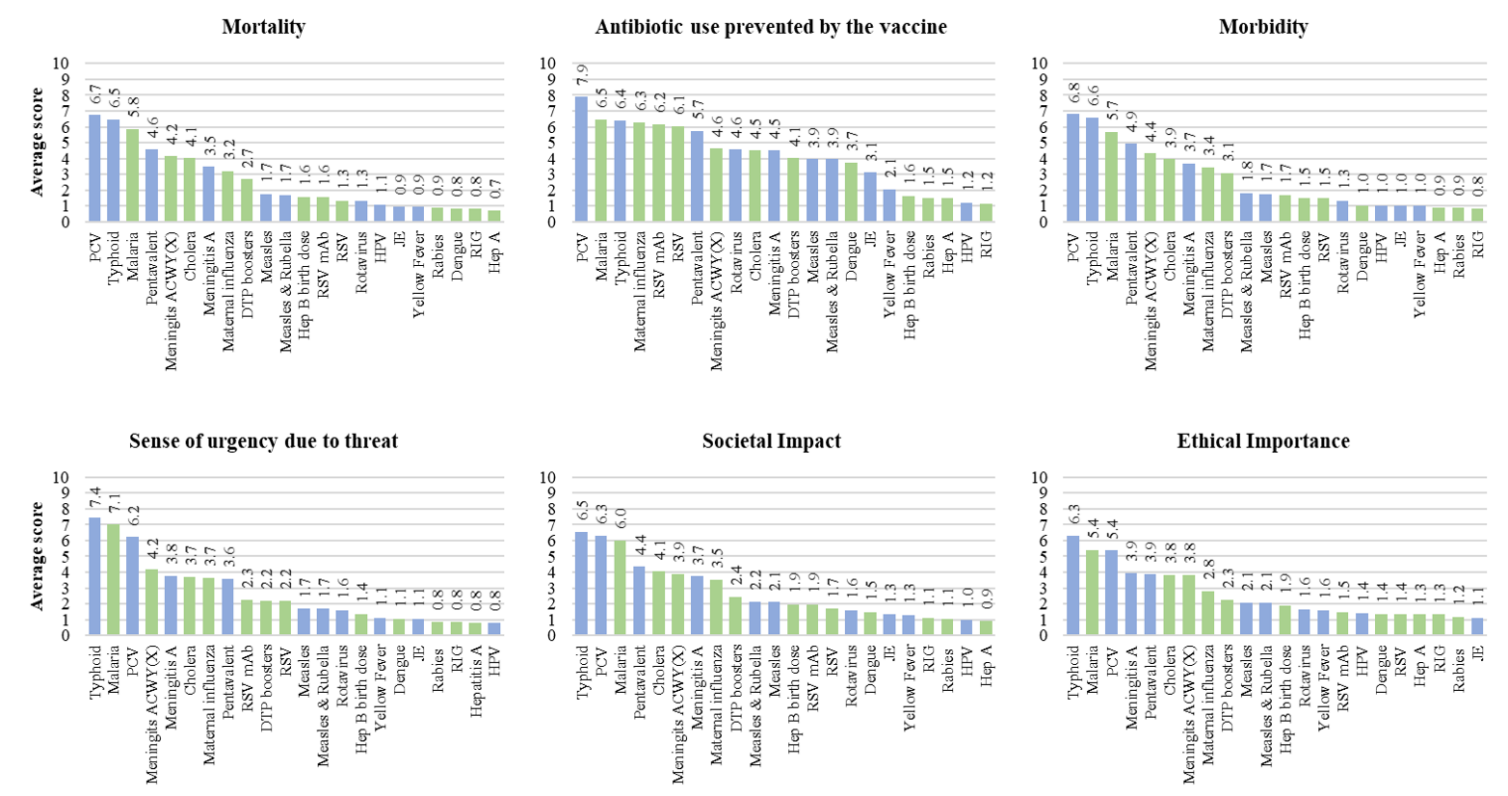
Vaccine impact on AMR, for all criteria, as estimated by experts. *(where 1= least effect of vaccine in future and 10 = greatest effect of vaccine in future) Blue shading = Gavi portfolio vaccines; Green shading = VIS candidate vaccines; numbers above the bars indicate mean criterion scores for each vaccine. PCV – pneumococcal conjugate vaccine; Pentavalent vaccine (diphtheria, pertussis, tetanus, hepatitis B and Haemophilus influenzae type B); DTP – diphtheria, tetanus & pertussis; RSV – Respiratory syncytial virus; mAb - monoclonal antibodies; Hep B – Hepatitis B; JE – Japanese encephalitis; HPV – Human papillomavirus; Hep A – Hepatitis A; RIG – Rabies immunoglobulin.*

More detailed scores for each criterion are presented in Figure 3. Across the criteria, similar trends were observed in terms of how experts perceived specific vaccines, suggesting a lack of independence between the criteria. Pneumococcal conjugate, malaria and typhoid vaccines were consistently attributed the highest impact scores across all criteria. Intermediate impact across most criteria were estimated for rotavirus, cholera, influenza, dengue, measles, meningitis, pentavalent vaccines and for diphtheria-tetanus-pertussis- (DTP-) containing vaccine boosters, as well as vaccines and monoclonal antibodies against RSV. Across criteria, lower values were attributed to Japanese encephalitis, hepatitis, yellow fever, rabies and HPV-related vaccines.

All results and anonymised participants information are available as underlying data.

## Discussion

While the fight against AMR has been identified as a major public health priority, the value of vaccines in contributing to the control of AMR has been difficult to articulate ^7– 9^. Vaccines can contribute to the control of AMR through various, complex mechanisms, and the estimation of the full value of investments for new interventions requires defining value across multiple preference metrics ^22^.

The results from this expert survey highlight the importance of investments in pneumococcal conjugate, malaria and typhoid vaccines, relative to other investments considered by Gavi, when their impact on AMR infections is taken into account.

Of these, Gavi developed an Advanced Market Commitment for pneumococcal conjugate vaccine (PCV) in 2009. To date 60 countries have introduced the vaccine into their national schedules with support from Gavi. An unpublished evaluation from the Gavi secretariat in collaboration with John Hopkins University assessed that past Gavi support has averted 14 million antibiotic doses for pneumonia between 2011–15 alone. For 2016–2020, Gavi has committed to vaccinate hundreds of millions of children with vaccines against meningitis and pneumonia, estimated to potentially prevent over 100 million further antibiotic doses (personal communication Hope Johnson). The evaluation indicates that the perceived impact of PCV on AMR is related primarily to the vaccine’s demonstrated potential to reduce antibiotic consumption. This supports continued efforts to increase coverage and global use of PCV.

Gavi funding for typhoid conjugate vaccine (TCV) was made available at the end of 2017 with the first introductions planned for 2019, including in Zimbabwe. Pakistan carried out a campaign with Vi-polysaccharide typhoid vaccine in 2017 in response to a growing number of extensively drug resistant cases of Salmonella typhi. To date, 118,000 children aged between 6 months and 10 years have been vaccinated ^23^. The specific role of TCV in the containment of resistant typhoid strains, and their utility in response to outbreaks with resistant strains should be further evaluated ^24^.

Gavi is co-funding the RTS,S/AS01 malaria vaccine implementation pilot evaluation in Ghana, Kenya and Malawi ^25^. Artemisinin-based combination therapies are recommended by WHO to treat uncomplicated *Plasmodium falciparum* malaria. Artemisinin-resistant malaria strains are present in South-East Asia. There is significant potential for spread, putting present malaria control strategies at risk ^26^. The possible future wide-scale implementation of the malaria vaccine RTS,S/AS01 in young African children is unlikely to play a major role in the containment of artemisinin-resistant malaria strains initially, but the contribution to reduction in antibiotic use associated to febrile episodes may be significant ^27^. Investigators are considering vaccine use in South-East Asia for the specific purpose of containing the spread of artemisinin resistance ^28^.

As could be expected, vaccination strategies targeting bacterial pathogens generally received high scores for impact on AMR-related mortality, morbidity and sense of urgency. Experts also attributed significant value to some vaccines targeting viral pathogens when considering reduction in antibiotic use. Altogether investments in vaccines protecting against rotavirus, cholera, RSV, influenza, dengue, measles and meningitis, as well as the Hib-containing pentavalent vaccine were also considered to be of value, – more so than those related to Japanese encephalitis, hepatitis, yellow fever, rabies and HPV vaccines.

Our results are broadly consistent with previous assessments of the value of vaccines in preventing AMR infections, including during a meeting held by the Chatham House in March 2017 ^7, 8^. The meeting identified several gaps, including developing an action framework for the use of vaccines to help contain AMR, gathering evidence and promoting research and development (R&D) of vaccines that could reduce antibiotic use and that protect against pathogens which are considered to be driving AMR threats. A poll was taken at the meeting to prioritise vaccines by their impact on AMR. Meeting participants identified tuberculosis, typhoid and influenza as the top-three priority vaccines for AMR impact ^8^.

A recent analysis conducted by Wellcome and the Boston Consulting Group investigated the role of vaccines in reducing AMR, targeting pathogens included in the WHO pathogen priority list of antibiotic-resistant bacteria ^4^ as well as *Mycobacterium tuberculosis*. Health impact, probability of R&D success, and probability of uptake were considered for 18 priority pathogen groups. The report emphasised the need to increase the uptake of vaccines currently in use (PCV, *Salmonella typhi* and Hib), and accelerate market entry of vaccines for shigella, non-typhoidal salmonella, and *Escherichia Coli* ^29^.

Lastly, the World Organisation for Animal Health (OIE) has conducted a review on prioritisation of diseases for which vaccines could reduce antimicrobial use in cattle, sheep and goats ^30^. Taking into consideration the antibiotic use associated with diseases and current vaccine availability, the OIE orders vaccine research priorities for a number of vaccines targeting specific animal pathogens ^30^.

Several limitations of this value attribution exercise need to be considered. It was launched to contribute to the VIS 2018 exercise with significant time constraints. To address the lack of data on AMR, and data on vaccine impact on AMR pathogens, we decided to seek an expert opinion to complete this exercise. A total of 18 experts completed the survey, but a higher number would have been desirable. Typically, for multi-criteria decision analyses, criteria should be complete, non-redundant, non-overlapping and independent ^31^. Some of these requirements were probably not completely met, and it is likely that mortality and morbidity outcomes influenced the expert assessment of sense of urgency, ethical importance and societal impact. The general homogeneity in the way specific vaccine investment were valued across different criteria suggests a lack of independence between criteria. It is also the case that some important considerations were not included in the value attribution framework, such as the availability of alternative interventions to control the pathogens considered. Despite its drawbacks, the exercise should be viewed as a preliminary attempt to estimate the comparative value of a range of vaccines in terms of their contribution to AMR control, and as a relevant benchmark for developing improved value attribution methodologies.

In the future, a more evidence-based, data-driven, robust methodology should be developed to assess the value of vaccines as a tool to control AMR, building on this exercise as well as the work conducted by Wellcome and Chatham House ^8, 29^. Complex modelling methods will likely have to be developed ^22^. A long-term perspective should be adopted, as the public health need is likely to continue to be important. Confronted by data gaps, error margins are predicted to be large. However, as there is growing impetus for research in this area, more evidence will emerge and there will be opportunities to refine estimates. Future exercises should consider an expanded list of pathogens including tuberculosis, enteric pathogens and group A and B streptococcus, as well as other vaccines that were not part of the Gavi VIS 2018.

There is a need to define and align on priority actions to strengthen the use of vaccines to control AMR and coordinate their implementation. This could include the expression of priorities in evidence generation, such as advocating collection and analysis of antibiotic consumption data in vaccine studies, reaching consensus on design and implementation of specific studies aimed at characterising the role of specific vaccines against AMR or determining how to better use data from bacterial disease surveillance networks and routine monitoring of vaccine use. Also for further consideration is the systematic incorporation of AMR impact in vaccine-related decision-making in industry, financing bodies, regulatory and policy forums, including the creation of incentives for the development of future vaccines or better use of available vaccines with impact on AMR. Both WHO and Gavi have an important role in supporting coordinated action and rational decision-making in vaccine research and implementation.

## Ethical approval and consent

This research is exempt from ethical approval. It involves the use of non-sensitive and anonymous surveys and interview procedures with participants that are not defined as “vulnerable” and participation will not induce undue psychological stress or anxiety. All participants gave a written consent to participate in the study and all data was anonymised prior to submission.

## Data availability

### Underlying data

Figshare: AMR GAVI VIS underlying data. https://doi.org/10.6084/m9.figshare.9724229 ^33^

This project contains the following underlying data:

- Raw data.xlsx (This is a raw data file with individual answers from study participants)

### Extended data

Figshare: AMR GAVI VIS Extended data. https://doi.org/10.6084/m9.figshare.9232115 ^32^

This project contains the following extended data:

- Background info for authors.xlsx (The excel file that contains the background information that was sent to all participants in the study; the questionnaire that asked participants to enter the vaccine and criteria scores; and participants anonymised details)
- Email body.pdf (The body of an email that was sent to all participants in the study)

Data are available under the terms of the Creative Commons Zero "No rights reserved" data waiver (CC0 1.0 Public domain dedication).
