# Supplementary material for: Vaccine impact on antimicrobial resistance to inform Gavi, the Vaccine Alliance’s 2018 Vaccine Investment Strategy: report from an expert survey
Source: F1000Res. 2019 Sep 24;8:1685. [Version 1] doi: 10.12688/f1000research.20100.1 (PMC6807152; doi:10.12688/f1000research.20100.1)
Supplement: Peer review history

# Peer review: Reviewer response for version 1

**DOI:** 10.5256/f1000research.22068.r54549

**Published:** 2019-10-21

**Authors:** David M. Salisbury

**Affiliations:** 1 Centre on Global Health Security, Chatham House, London, UK

This study takes a number of infections for which vaccine interventions exist or may exist, considers their relevance to AMR and takes into account the vaccines that have been prioritised for investment by GAVI. A number of  experts have been asked to prioritise these vaccines against criteria that were predetermined and scored against AMR importance elements. This prioritisation exercise is very similar to those previously undertaken by and on behalf of the WHO Strategic Advisory Group of Experts on immunisation (SAGE). The results of these previous exercises, as this one, were driven essentially by mortality, and to a lesser degree, by morbidity: the results are not surprising irrespective of the complexity of the scoring system. Meanwhile, some confidence can be derived from this subjective process that the GAVI vaccine priorities remain relevant from an AMR perspective.

I confirm that I have read this submission and believe that I have an appropriate level of expertise to confirm that it is of an acceptable scientific standard.

# Peer review: Reviewer response for version 1

**DOI:** 10.5256/f1000research.22068.r54296

**Published:** 2019-10-01

**Authors:** William Hausdorff

**Affiliations:** 1 Faculty of Medicine, Free University of Brussels (ULB), Brussels, Belgium; 2 PATH, Washington DC, USA

Based on a survey of 18 global experts in antimicrobial resistance, this article recounts their perceptions of the value relative to AMR of investments in specific vaccines currently underway or being considered by GAVI.  As such, it provides 2 types of information that are  valuable: 1) expert views of the relative importance of pre-defined criteria by which to assess vaccine value vis a vis AMR and 2) expert assessments of specific vaccines.

 The paper is nicely written, but there are a few places where greater clarity would help make interpretation of the results more straightforward.  

 Specifically, I remain unclear whether experts were assessing the vaccines based on their **potential **value or based on their **current **value.  For example, current pneumococcal conjugate vaccines, while they all but eliminate antimicrobial non-susceptible invasive disease caused by 10-13 vaccine serotypes, don't prevent such disease caused by the other 80+ non-vaccine serotypes - and many of those can be antibiotic non-susceptible.  Is the high valuation of PCVs based on the current PCVs or on a future common antigen vaccine/25+ valent conjugate?  Similarly, the current RTS,S vaccine has real but limited efficacy - is the high valuation of malaria due to this vaccine or only to a theoretically 95% effective vaccine?

 Secondly, it was unclear to me whether the high priority given to a vaccine's ability to prevent  mortality and morbidity was related to **overall** disease burden caused by that pathogen, or the magnitude of the **antimicrobial non-susceptible** disease burden caused by that pathogen, or the **potential antimicrobial non-susceptible** disease burden.  This would help in interpreting the relatively high priority given to Hib-containing pentavalent vaccine, for example.  And I'm not sure how much pneumococcal mortality is currently due to antimicrobial resistant pneumococci.

 If the answer is that no further clarity is possible due to ambiguous wording of the questions, then such ambiguity could be pointed out as something to avoid in the future.

 An additional limitation that should be highlighted is that all of the 18 experts queried appear to be from high income countries, with the exception of one (or more?) Indian expert.

 It was unclear whether the outlier data points in Figures 1 and 2 were included in the calculations of the medians and means.  It seems a bit strange that the error bars said to denote minimum and maximum values do not include those outliers; that means the error bars should be more accurately described as a CI covering X% of the data.

 More minor comments to address to improve clarity:

1. Page 3, Background, mentions "a similar post-introduction study in South Africa" of PCV, but the specific results described for that study do not provide any evidence of impact against AMR.
2. Page 8, first few lines would seem to imply that 118,000 children mentioned all received typhoid polysaccharide vaccine, but in reality they received typhoid conjugate vaccine.
3. Also on page 8, it is unclear to me how the Chatham House prioritization of tuberculosis, typhoid, and influenza is "broadly consistent" with the results presented here.

I confirm that I have read this submission and believe that I have an appropriate level of expertise to confirm that it is of an acceptable scientific standard.
